# Living with chronic rhinosinusitis with nasal polyposis (CRSwNP): an experience to amplify Italian patients’ voices

**DOI:** 10.1186/s41687-025-00990-2

**Published:** 2026-01-30

**Authors:** L. Bernardi, M. R. Mollo, C. Sartor, S. Barbaglia, D. Gargano, F. Anastasi, G. Camiciottoli, C. Allegrini, C. Caruso, I. Baiardini, L. Cappellari

**Affiliations:** 1 GSK Medical Department, Viale dell’Agricoltura, 7, Verona, Italy; 2 National Patient Association Respiriamo Insieme aps, Via Niccolò Tommaseo 94, 35131 Padova, Italia; 3 U.O. Allergologia e Immunologia Clinica, Azienda Ospedaliera di Rilievo Nazionale e di Alta Specialità San Giuseppe Moscati, 83100 Avellino, Italia; 4 Unit of Otorhinolaryngology, Medical and Surgical Rhinology, Ospedale San Giovanni Evangelista, Tivoli, Italy; 5 Facoltà Dipartimentale di Medicina, UniCamillus—Saint Camillus International University of Health and Medical Sciences, Roma, Italy; 6 Scienze Biomediche Sperimentali e Cliniche “Mario Serio”, Università degli Studi di Firenze, P.za di San Marco, 4, Firenze, Italia; 7 Pneumologia e Fisiopatologia Toraco-Polmonare, Azienda Ospedaliera Unviersitaria Careggi, Largo Giovanni Alessandro Brambilla, 3, Firenze, Italia; 8 Allergy and Clinical Immunology Unit, Fondazione Policlinico A. Gemelli IRCCS, Largo Agostino Gemelli, 8, Roma, Italia; 9 Department of Health and Life Science, European University of Rome, Roma, Italia

**Keywords:** Patient engagement, Patient advocacy, Patient empowerment, Chronic rhinosinusitis with nasal polyposis

## Abstract

**Background:**

Chronic rhinosinusitis with nasal polyps (CRSwNP) significantly impacts patients’ quality of life, yet there is a notable lack of studies addressing the disease burden and patients’ perspectives compared to other conditions such as asthma and allergies. This project aimed to gather insights from both patients living with CRSwNP and healthcare professionals (HCPs) involved in its management, with the goal of better understanding their experiences and identifying shared unmet needs and expectations.

**Method:**

Twelve CRSwNP patients participated in2 focus groups (open-ended questions) facilitated by a moderator. The insights gathered were presented to an expert board comprising HCPs and expert patients, both of whom are members of the PAG. Patients involved in the expert board enriched the qualitative data collection providing narratives of their personal experience.

**Results:**

A total of 24 patients with CRSwNP participated in the analysis, 124 qualitative insights, distinct observations, comments or experiences related to their conditions, were gathered. The main priorities expressed were to decrease invasive procedures; avoid chronicity/reduce relapses; and create more opportunities for patients to be heard.

**Conclusion:**

Participants expressed expectations that extended beyond disease management, emphasizing the importance of their active involvement in shaping healthcare strategies. This project highlights the critical need to actively listen to the experiences and unmet needs of both patients and HCPs, integrating their perspectives into healthcare strategies to deliver practical, patient-centered improvements in the management of CRSwNP.

**Supplementary Information:**

The online version contains supplementary material available at 10.1186/s41687-025-00990-2.

## Key summary points

While CRSwNP significantly affects patients’ quality of life, there is a notable lack of published studies exploring the disease burden and patients’ perspectives, especially when compared to other conditions such as asthma and allergies.

This article explores the daily challenges and emotional impact experienced by Italian patients with CRSwNP, emphasizing the need for more empathetic, patient-centered approaches to care.

The diverse experiences of these patients highlight the importance of considering cultural and regional factors in the management of CRSwNP, underscoring the need for future research and healthcare improvements that are tailored to patients’ specific needs.

**Supplementary Information:**

The online version contains supplementary material available at 10.1186/s41687-025-00990-2.

## Introduction

Chronic rhinosinusitis (CRS) is a persistent inflammatory condition that affects the nose, paranasal sinuses, and upper airways. It is characterized by the presence of specific symptoms, including nasal blockage, congestion, nasal discharge, facial pain or pressure, and a reduced sense of smell, which persist for at least 12 weeks [1, 2]. CRS can be further classified into chronic rhinosinusitis without nasal polyps (CRSsNP) and chronic rhinosinusitis with nasal polyps (CRSwNP), the latter being associated with bilateral polyps [3–5].

Studies have indicated that the prevalence of CRSwNP is 1–2% in Europe, significantly impacting patients’ quality of life [6]. The disease is often associated with comorbidities such as asthma, which is present in 10% to 60% of cases, leading to increased disease severity [7]. 

Although the direct and indirect costs of patients with CRSwNP in Europe remain largely unexplored, a study in the United States revealed a substantial annual healthcare cost of $5.7 billion for these patients, highlighting the significant financial burden associated with the condition [8, 9].

Patients with CRSwNP experience significantly reduced health-related quality of life (QoL) compared with healthy individuals. The impact is more pronounced in patients with greater disease severity, comorbidities, or refractory disease [10]. Symptoms associated with nasal polyps often affect mental and physical health, social functioning, sleep, and even workplace performance [11, 12].

Patient-reported outcome measures (PROMs) have been developed to quantify disease impact, including the Sino-Nasal Outcome Test (SNOT-22) [13], the Rhinosinusitis Disability Index (RSDI) [14], and the Nasal Obstruction Symptom Evaluation (NOSE) scale [15]. While these tools provide valuable quantitative insights, they may not fully capture the nuances of patient experiences.

Few qualitative studies on patients’ experiences and perspectives of current management of CRSwNP have been published [16]. Some of these studies have explored different aspects of patient experiences: unmet needs [17], the overall patient journey [18], and patient views of current management in primary and secondary care [19]. However, studies focusing on the Italian patient experience remain scarce. In addition, increasing importance has been placed on patient-reported outcomes (PROs) and QoL improvements by EPOS/EUFOREA experts in evaluating both disease control and the efficacy of treatments [6].

The present project aimed to bridge this gap by collecting insights directly from Italian patients with CRSwNP. Insights included patient’ experiences, observations, and comments related to their symptoms, treatment, and daily life. By actively listening to their experiences, the project sought to understand the patient journey from symptom onset to diagnosis and treatment, with a focus on the impact of symptoms on daily life. By aligning these patient-centric perspectives with those of HCPs, the project aimed to identify common unmet needs and expectations among patients with CRSwNP.

## Methodology

Two separate patient focus groups were conducted, involving a total of 12 Italian subjects diagnosed with CRSwNP. Participants were recruited on a voluntary basis by healthcare professionals (HCPs) and the patient advocacy group (PAG) Respiriamo Insieme APS. Recruitment was opportunistic, aiming to engage patients who were willing and able to participate. During these sessions, patients were asked open-ended, predefined questions in an interactive manner facilitated by a moderator. The questions focused on various aspects of the patient’s journey, including disease onset, diagnosis, the impact of the disease on QoL, expectations regarding treatments and expectations of different stakeholders, such as institutions, HCPs, PAGs, and pharma industry. The topics and questions for the focus groups were predetermined and can be found in Appendix 1. The primary objective of the focus groups was to gather valuable insights.

In the field of psychotherapy, insight is defined as the awareness of underlying sources of emotional, cognitive, or behavioural responses and difficulties in oneself or others [20]. Insight can be defined not only in terms of people’s understanding of their illness but also in terms of understanding how the illness affects their interactions with the world [21]. 

Accordingly, in this collaborative project, we defined the concept of “insight” to encompass the perspectives that emerge from the interaction and collaboration between those expressing it and those collecting/acquiring it. It aims to explain the motivations, such as needs, doubts, obstacles, and emotions, that underlie specific behaviours and actions. The collection of insights helps to better understand the deep needs, barriers, and desires of patients, thereby identifying potential actions and practical changes to meet their needs.

The insights collected during the focus groups were the basis to drive a discussion with an expert board consisting of representatives from the PAG Respiriamo Insieme APS and HCPs who were members of the association’s scientific board. The expert board comprised 12 patients from Respiriamo Insieme APS, all of whom had significant experience with CRSwNP, and 6 HCPs (2 allergists; 1 ear, nose and throat specialist (ENT); 2 pulmonologists; and 1 psychologist). The identified participants were divided into 2 separate expert focus groups, moderated by an experienced facilitator. The expert focus groups had preestablished topics and questions to facilitate discussions on the insights generated from the patient focus groups. CRSwNP patient participating to the expert board were asked to also provide insights on their personal experience in a dedicated open-question discussion. The goal of these expert board meetings was to reach a consensus on the identification of shared needs among patients with CRSwNP and HCPs.

**Fig. 1:**
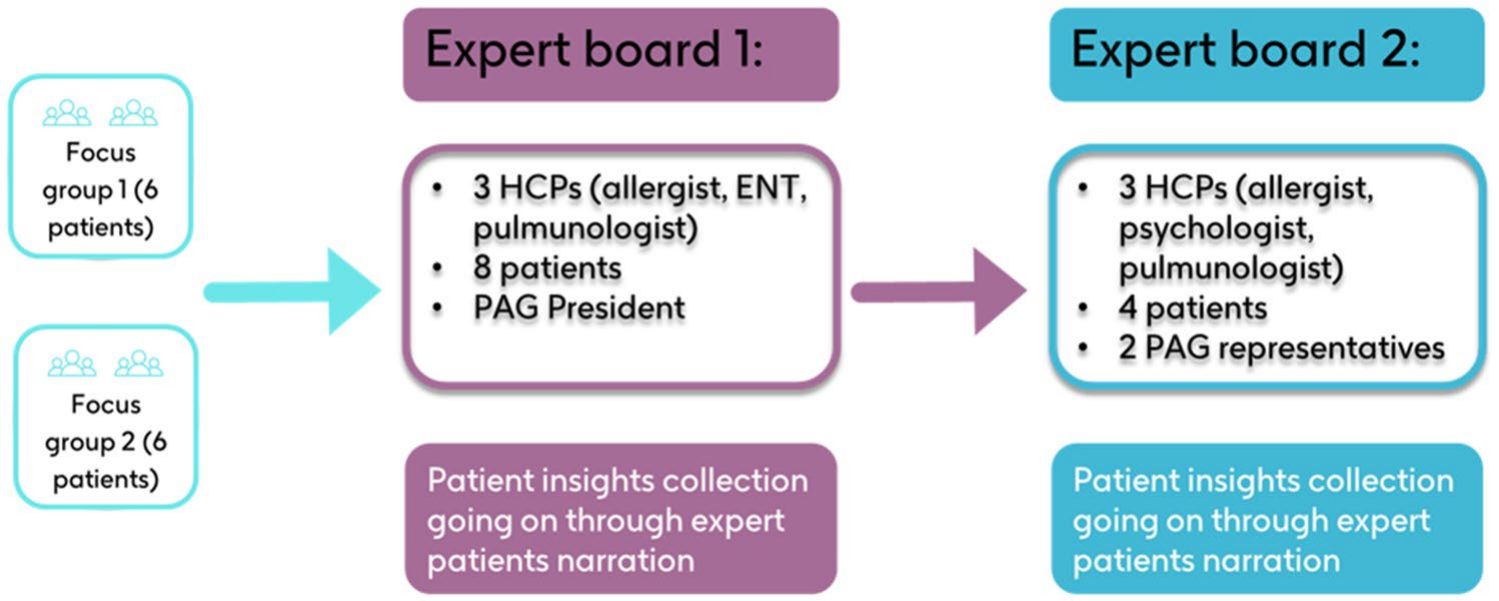
Schematic flow of the project

Figure 1 provides a schematic flow of the project, illustrating the steps followed.

Few empirical studies exist to guide researchers in determining the number of focus groups necessary for a research study. An analysis from Guest et al. revealed that more than 80% of all themes were discoverable within two to three focus groups, and 90% were discoverable within three to six focus groups [22]. 

Notably, the project followed a focus group methodology, with the questions intended to guide a broader discussion among participants rather than being answered in an interview-like format [23]. 

This project did not require IRB approval because is framed as a patient engagement activity, with a collaborative nature.

## Results

A total of 124 insights were gathered from 24 patients with CRSwNP (see Table 1 for demographic details). The key themes identified are presented in the following sections.

**Table 1:** Demographics of the patients included in the project

	Sample (*N* = 24)
Female (%)	42% (*N* = 10)
Male (%)	58% (*N* = 14)
Age (range)​	28–70​
Age (mean)	49

### Impact of symptoms on quality of life

The patient focus groups highlighted several key burdens associated with CRSwNP, emphasizing the substantial impact of symptoms on various dimensions of quality of life (QoL). While the focus group questions did not explicitly inquire about the effects of symptoms on occupational or educational activities, the discussions revealed that these domains were significantly affected. Below are selected examples of patients’ responses:

**Table:** 

*Quote*	*Gender*	*Age group*
*“I could no longer smell anything*,* and it was dangerous. I could not even smell anything important for safety*,* such as gas leaks.”*	*Female*	*31–50 years*
*“I had difficulty eating and could only consume liquids.”*	*Female*	*31–50 years*
*“I always had colds and headaches. I could not be efficient when I went to school.”*	*Female*	*16–30 years*
*“My problem was coughing. I am a teacher*,* and during class*,* I had become coughphobic.”*	*Female*	*31–50 years*

*Representative Patient Quotes*

### Late diagnosis

The primary symptoms driving patients to pursue further medical evaluation were severe pain and persistent cough. Notably, none of the participants reported receiving a recommendation from their general practitioner (GP) to consult a specialist in otolaryngology (ENT). Instead, the decision to seek specialized care was frequently influenced by advice from friends, family members, or other professionals within their social or professional networks. Below are selected examples of patients’ responses:

**Table:** 

*Quote*	*Gender*	*Age group*
*“I always suffer from colds and headaches. I could not be efficient when I went to school; I was always cold. My problem was always underestimated. I did not want to be misunderstood; it was kind of underestimated by everyone.”*	*Female*	*16–30 years*
*“I have been around allergists*,* otolaryngologists*,* and pulmonologists for years*,* and the diagnosis has always been the same vasomotor rhinitis. I always take the same medications*,* such as cortisone and nasal sprays*,* sometimes even antibiotics. It was a constant wandering without any answers.”*	*Female*	*31–50 years*
*“I met at least 20 doctors scattered all over Italy and always everything privately but never arrived at a definitive cure.”*	*Male*	*31–50 years*

*Representative Patient Quotes*

### Psychological impact of surgery

All participants had undergone surgical intervention after the failure of initial medical treatments. Surgery was described by patients as a “last resort” and a necessary measure to achieve improvement in their condition. However, the possibility of requiring additional surgical procedures was perceived as psychologically “demoralizing.” Participants also reported experiencing feelings of discouragement and apprehension related to postoperative care and recovery. Below are selected responses from participants:

**Table:** 

*Quote*	*Gender*	*Age group*
*“I don’t want to have surgeries anymore. I have had 5 of them*,* and the pain of postoperative swabs terrifies me.”*	*Female*	*31–50 years*
*“For me*,* it was devastating. I want drug therapy to replace surgery.”*	*Male*	*31–50 years*
*“Surgery for me is the last chance.”*	*Female*	*31–50 years*
*“We need faster*,* less invasive surgery with less hospital stay.”*	*Male*	*31–50 years*

*Representative Patient Quotes*

### Expert focus groups highlight the need for integrated action

**Fig. 2:**
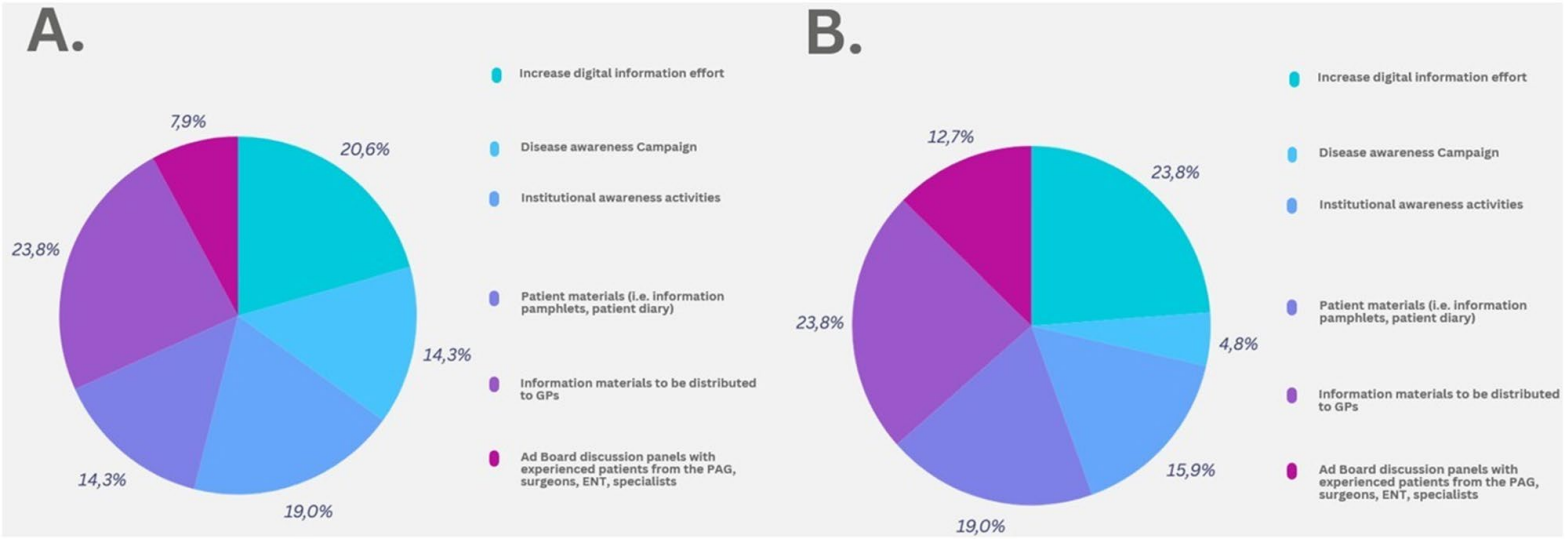
Actions identification and prioritization. **A**. Actions prioritization according to HCPs. **B**. Actions prioritization according to patient representatives (PAG). Labels and percentages on chart

The insights validated by the experts in the two dedicated focus groups analysing patients’ results highlight key areas for improvement that are summarized in Table 2 and further explained in the next paragraphs (See prioritized action in Fig. 2).

**Table 2:**
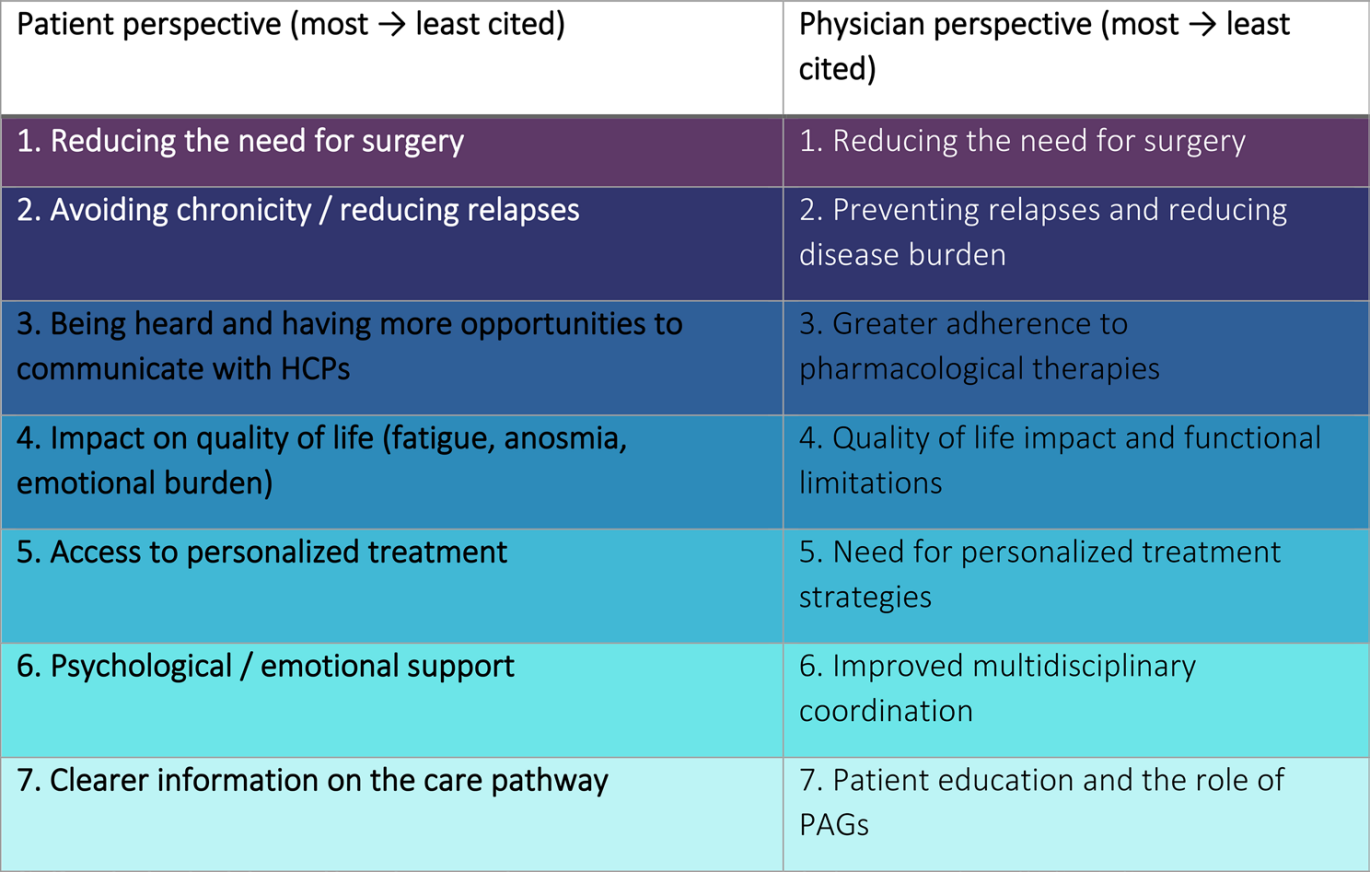
Summary of the shortcomings identified from the expert focus group meetings

Data derived from patient and clinician expert focus groups. Items are ordered from most to least frequently mentioned, highlighting shared priorities and unmet needs in CRSwNP care

#### Institutional initiatives for enhanced early disease identification and standardization of healthcare pathways

Institutional initiatives were deemed essential for improving early disease identification and promoting standardized healthcare pathways at the national level in Italy. A notable lack of educational resources for patients and clear diagnostic and therapeutic pathways was identified, particularly for individuals with CRSwNP. Consequently, a call to action was directed at institutions to emphasize the social and economic impact of this condition. Proper recognition of the disease, the establishment of definitive diagnostic and therapeutic pathways, the provision of educational opportunities, and the reduction of regional disparities in healthcare delivery were identified as critical priorities.

#### Advancing patient education and information dissemination efforts

The expert focus groups underscored the need to enhance patient education and improve the dissemination of informational materials, including through digital platforms. Additionally, the importance of training HCPs—particularly GPs and ENTs—and fostering networking opportunities among specialists was highlighted as a means to improve patient care.

#### Implementation of multidisciplinary disease management and holistic patient care

The discussions emphasized the importance of adopting a multidisciplinary approach to disease management and fostering a holistic model of patient care. Inadequate communication among specialists was identified as a potential contributor to inaccurate diagnoses and the development of additional comorbidities, such as adverse effects from prolonged corticosteroid use. Furthermore, the need to strengthen connections between hospitals and local healthcare services was highlighted.

#### Increased patient advocacy through inclusion in decision-making processes

The initiative was widely appreciated, as there is an enormous need among patients to share their perspective because of their current underrepresentation in decision-making discussions.

## Discussion

This project sought to explore the perspectives of patients diagnosed with chronic rhinosinusitis with nasal polyps (CRSwNP) through the facilitation of focus groups involving both patients and clinical experts. The primary objective was to engage all relevant stakeholders in the management and decision-making processes for CRSwNP, with a particular emphasis on enhancing patients’ quality of life (QoL). While the project was not designed to represent the entirety of the CRSwNP patient population, the sample size was informed by prior qualitative research methodologies, which suggest that a cohort of approximately ten participants, coupled with three to four focus group sessions, is sufficient to capture a diverse range of perspectives without compromising the depth or coherence of the findings [24].

Initially conceptualized as a patient engagement initiative rather than a formal research study, this project yielded valuable insights into the unmet needs and lived experiences of individuals with CRSwNP. Participants were recruited voluntarily through healthcare professionals (HCPs) and the patient advocacy organization Respiriamo Insieme APS. The focus groups were facilitated by individuals with medical expertise and prior experience in conducting structured discussions. Although the facilitators were unfamiliar to the participants, the sessions were designed to encourage open dialogue, with small group sizes and a duration of approximately two hours per session to ensure equitable participation. Discussions were guided by predefined questions, and the qualitative data collected were transcribed and categorized into overarching thematic areas. The project was not intended to achieve statistical significance or generalizability; rather, its primary aim was to explore patient perspectives and identify critical unmet needs. Despite limitations in reporting methodological rigor—such as data saturation, coding processes, and participant feedback—the findings underscore the importance of addressing patient needs through a multidisciplinary and patient-centered approach to CRSwNP care.

Additionally, it is noteworthy that a similar initiative was recently conducted by EUFOREA, an international nonprofit organization that collaborates with stakeholders from national and international entities. The findings from the EUFOREA study, published in 2021, highlighted the underestimation of the burden of CRSwNP and identified gaps in current care practices, offering actionable recommendations for improving the management of this debilitating condition (Table 3) [16].

**Table 3:**
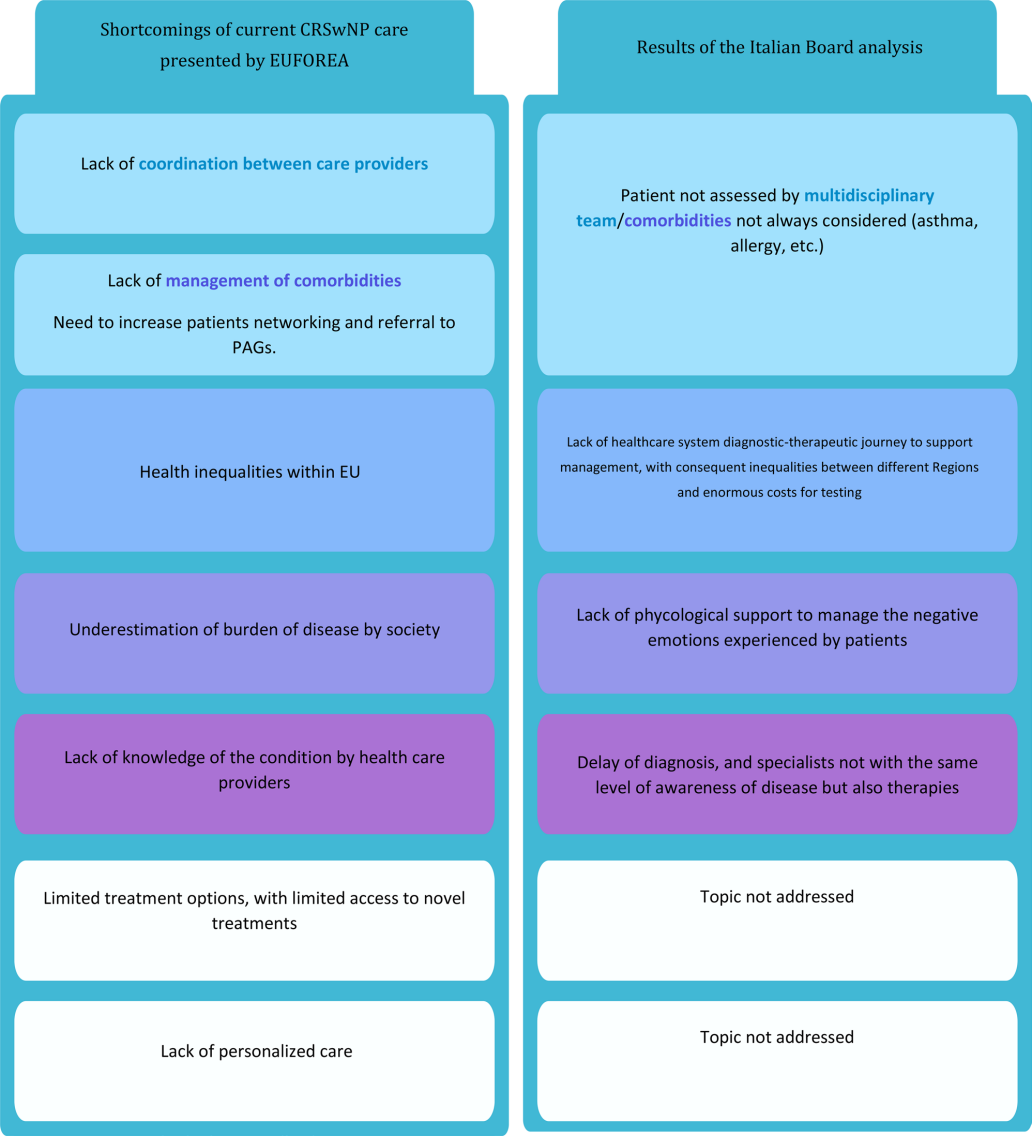
Comparison of the results of the EUFOREA board and Italian board

As evident from Table 3 the results of this project closely align with the conclusions of the EUFOREA board, which is particularly significant given the absence of Italian patients in the EUFOREA study cohort.

A patient-centered approach promotes enhanced communication between HCPs and patients, offering additional opportunities for patients to express their concerns and actively participate in their care. By considering patients’ preferences and treatment objectives, this approach aims to increase patient satisfaction and treatment outcomes. Empowering patients to take a more active role in their healthcare fosters a collaborative and trusting relationship between patients and HCPs, resulting in more personalized and effective interventions tailored to individual needs [25].

The project was not designed to achieve statistical significance or generalizability, as the primary aim was to explore patient perspectives and identify key unmet needs. Data were self-reported, which may introduce self-reporting bias, and formal methodological procedures such as detailed coding processes or participant validation were not undertaken, potentially limiting replicability. Despite these limitations, the findings highlight key areas for improvement in CRSwNP care, provide valuable insights into patient priorities and unmet needs, and suggest that future projects could adopt structured reporting to strengthen transparency and rigor.

The priorities identified by this Italian project, as reducing the need for surgery, preventing chronicity, and strengthening patient–physician communication, are likely to be relevant across different healthcare systems and cultural contexts. These findings therefore support the broader international dialogue on improving CRSwNP care and reinforce the importance of a patient-centered, multidisciplinary approach.

## Conclusion

Although the project was not designed to represent the entire CRSwNP patient population, the findings emphasize the importance of exploring patient perspectives to identify unmet needs and key challenges in CRSwNP care. Patients expressed expectations that extended beyond disease management, emphasizing the desire for active involvement in their care journey. This underscores the need to promote collaboration among pharmaceutical companies, patient advocacy organizations, healthcare professionals (HCPs), and institutional stakeholders. Such partnerships can facilitate the prioritization and implementation of actionable strategies driven by the patient voice, ultimately contributing to the advancement of care standards for individuals living with CRSwNP.

**Appendix 1:** Patient focus group questions

Questions
1. Could you describe the basic steps in the process leading to the diagnosis of nasal polyposis?
2. Who played a key role in referring you to the ENT doctor treating you?
3. For those of you who underwent cycles of systemic cortisone during the year, what particular consequences did you experience?
4. What aspects have had the greatest impact on the quality of your life? How did you have to change your lifestyle?
5. What are the most annoying symptoms that nasal polyps have caused you?
6. What do you wish to achieve further from surgical and/or medical treatment?
7. How did you experience the need for surgery and what impact did it have?
8. What would you expect from a new drug treatment to improve your current condition?

## Supplementary Information

Below is the link to the electronic supplementary material.

Supplementary Material 1

## Data Availability

The datasets generated during and/or analyzed during the current study are available from the corresponding author on reasonable request.

## Declarations

### Congresses

A poster regarding this publication was submitted, accepted and presented at SIAIIC 2023 (HOTEL SAVOIA REGENCY – Via del Pilastro 2–40127 Bologna; 27.10.2023).

## References

[1] Fokkens WJ, Lund V, Bachert C, Mullol J, Bjermer L, Bousquet J et al (2019) EUFOREA consensus on biologics for CRSwNP with or without asthma. Allergy 74:2312–2319. 10.1111/all.13875. 31090937 10.1111/all.13875 PMC6972984

[2] Schleimer RP (2017) Immunopathogenesis of chronic rhinosinusitis and nasal polyposis. Annu Rev Pathol Mech Dis 12:331–357. 10.1146/annurev-pathol-052016-100401. 10.1146/annurev-pathol-052016-100401 PMC5514544 27959637

[3] Fokkens WJ, Lund VJ, Hopkins C, Hellings PW, Kern R, Reitsma S et al (2020) European position paper on rhinosinusitis and nasal polyps 2020. Rhinology 0:1–464. 10.4193/Rhin20.600. 10.4193/Rhin20.600 32077450

[4] Bachert C, Pawankar R, Zhang L, Bunnag C, Fokkens WJ, Hamilos DL et al (2014) ICON: chronic rhinosinusitis. World Allergy Organ J 7:25. 10.1186/1939-4551-7-25. 25379119 10.1186/1939-4551-7-25 PMC4213581

[5] Hopkins C (2019) Chronic rhinosinusitis with nasal polyps. N Engl J Med 381:55–63. 10.1056/NEJMcp1800215. 31269366 10.1056/NEJMcp1800215

[6] Fokkens WJ, De Corso E, Backer V, Bernal-Sprekelsen M, Bjermer L, von Buchwald C et al (2024) EPOS2020/EUFOREA expert opinion on defining disease States and therapeutic goals in CRSwNP. Rhinology 0:0–0. 10.4193/Rhin23.415. 10.4193/Rhin23.415 38217529

[7] Canonica GW, Malvezzi L, Blasi F, Paggiaro P, Mantero M, Senna G et al (2020) Chronic rhinosinusitis with nasal polyps’ impact in severe asthma patients: evidences from the severe asthma network Italy (SANI) registry. Respir Med 166:105947. 10.1016/j.rmed.2020.105947. 32250875 10.1016/j.rmed.2020.105947

[8] Lourijsen ES, Fokkens WJ, Reitsma S (2020) Direct and indirect costs of adult patients with chronic rhinosinusitis with nasal polyps. Rhinology 0:0–0. 10.4193/Rhin19.468. 10.4193/Rhin19.468 32415826

[9] Bhattacharyya N, Villeneuve S, Joish VN, Amand C, Mannent L, Amin N et al (2019) Cost burden and resource utilization in patients with chronic rhinosinusitis and nasal polyps. Laryngoscope 129:1969–1975. 10.1002/lary.27852. 30720213 10.1002/lary.27852 PMC6767455

[10] Khan A, Huynh TMT, Vandeplas G, Joish VN, Mannent LP, Tomassen P et al (2019) The GALEN rhinosinusitis cohort: chronic rhinosinusitis with nasal polyps affects health-related quality of life. Rhinology 0:0–0. 10.4193/Rhin19.158. 10.4193/Rhin19.158 31318362

[11] Bachert C, Sousa AR, Lund VJ, Scadding GK, Gevaert P, Nasser S et al (2017) Reduced need for surgery in severe nasal polyposis with mepolizumab: randomized trial. J Allergy Clin Immunol 140:1024–31e14. 10.1016/j.jaci.2017.05.044. 28687232 10.1016/j.jaci.2017.05.044

[12] Alobid I, Benítez P, Bernal-Sprekelsen M, Roca J, Alonso J, Picado C et al (2005) Nasal polyposis and its impact on quality of life: comparison between the effects of medical and surgical treatments. Allergy 60:452–458. 10.1111/j.1398-9995.2005.00725.x. 15727575 10.1111/j.1398-9995.2005.00725.x

[13] Misirovs R et al (2024) 5-Item Sino-Nasal outcome test and 22-Item Sino-Nasal outcome test: A comparative study in chronic rhinosinusitis with nasal polyps. Ann Allergy Asthma Immunol 132(3):363–367. 10.1016/j.anai.2023.12.002. 37984707 10.1016/j.anai.2023.11.011

[14] Birch DS, Saleh HA, Wodehouse T, Simpson IN, Mackay IS (2001) Assessing the quality of life for patients with chronic rhinosinusitis using the rhinosinusitis disability index. Rhinology 39(4):191–196. 10.4193/Rhinology.2001.39.4.191. 11826687

[15] Stewart MG, Witsell DL, Smith TL, Weaver EM, Yueh B, Hannley MT (2004) Development and validation of the nasal obstruction symptom evaluation (NOSE) scale. Otolaryngol Head Neck Surg 130(2):157–163. 10.1016/j.otohns.2003.09.016. 14990910 10.1016/j.otohns.2003.09.016

[16] Claeys N, Teeling MT, Legrand P, Poppe M, Verschueren P, De Prins L et al (2021) Patients unmet needs in chronic rhinosinusitis with nasal polyps care: a patient advisory board statement of EUFOREA. Front Allergy. 2021;2:761388. 10.3389/falgy.2021.761388. 10.3389/falgy.2021.761388 PMC8974789 35386961

[17] Claeys N, Teeling MT, Legrand P, Poppe M, Verschueren P, De Prins L et al (2021) Patients’ unmet needs in chronic rhinosinusitis with nasal polyps: a European perspective. Front Allergy 2:761388. 10.3389/falgy.2021.761388. 35386961 10.3389/falgy.2021.761388 PMC8974789

[18] Hwee J et al (2024) The chronic rhinosinusitis with nasal polyp patient journey in the united States and Europe. Allergy Asthma Clin Immunol 20:17. 10.1186/s13223-024-00879-7. 38409099 10.1186/s13223-024-00879-7 PMC10898083

[19] Vennik J et al (2019) Chronic rhinosinusitis: a qualitative study of patient views and experiences of current management in primary and secondary care. BMJ Open 9:e025752. 10.1136/bmjopen-2018-025752. 10.1136/bmjopen-2018-022644 PMC6501991 31015263

[20] VandenBos GR (ed) (2015) APA dictionary of psychology, 2nd edn. Amer Psychol Assoc, Washington. 10.1037/14646-000.

[21] Marková IS, Berrios GE (1992) The meaning of insight in clinical psychiatry. Brit J Psychiatry 160:850–860. 10.1192/bjp.160.6.850. 1617369 10.1192/bjp.160.6.850

[22] Guest G, Namey E, McKenna K (2017) How many focus groups are enough? Building an evidence base for nonprobability sample sizes. Field Methods 29:3–22. 10.1177/1525822X16639015.

[23] Rosaline S. In: Dingwall R, de Vries R, editors. The SAGE handbook of qualitative methods in health research. London: SAGE Publications; 2010. p.

[24] Rabiee F (2004) Focus-group interview and data analysis. Proc Nutr Soc 63:655–660. 10.1079/PNS2004399. 15831139 10.1079/pns2004399

[25] Hellings PW, Fokkens WJ, Bachert C, Akdis CA, Bieber T, Agache I et al (2017) Positioning the principles of precision medicine in care pathways for allergic rhinitis and chronic rhinosinusitis – A-EUFOREA-ARIA‐EPOS‐AIRWAYS ICP statement. Allergy 72:1297–1305. 10.1111/all.13162. 28306159 10.1111/all.13162

