# Supplementary material for: Living with chronic rhinosinusitis with nasal polyposis (CRSwNP): an experience to amplify Italian patients’ voices
Source: J Patient Rep Outcomes. 2026 Jan 30;10:22. doi: 10.1186/s41687-025-00990-2 (PMC12901749; doi:10.1186/s41687-025-00990-2)
Supplement: Supplementary file 1 — Supplementary Material 1 [file 41687_2025_990_MOESM1_ESM.pptx]

## Slide 1
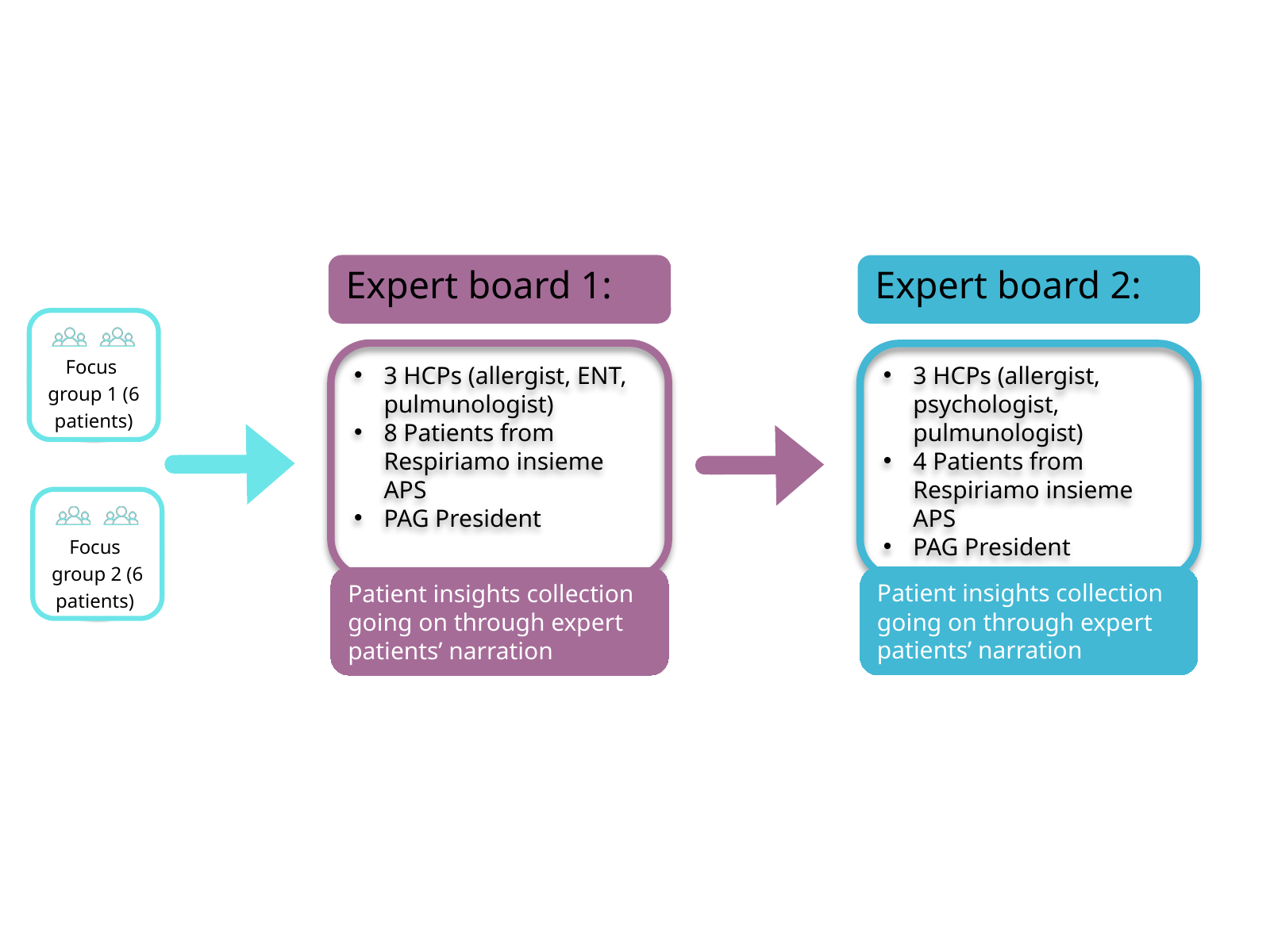

Expert board 1:
3 HCPs (allergist, ENT, pulmunologist)
8 Patients from Respiriamo insieme APS
PAG President
Patient insights collection going on through expert patients’ narration
Expert board 2:
3 HCPs (allergist, psychologist, pulmunologist)
4 Patients from Respiriamo insieme APS
PAG President
Patient insights collection going on through expert patients’ narration
Focus
group 1 (6 patients)
Focus
group 2 (6 patients)

## Slide 2
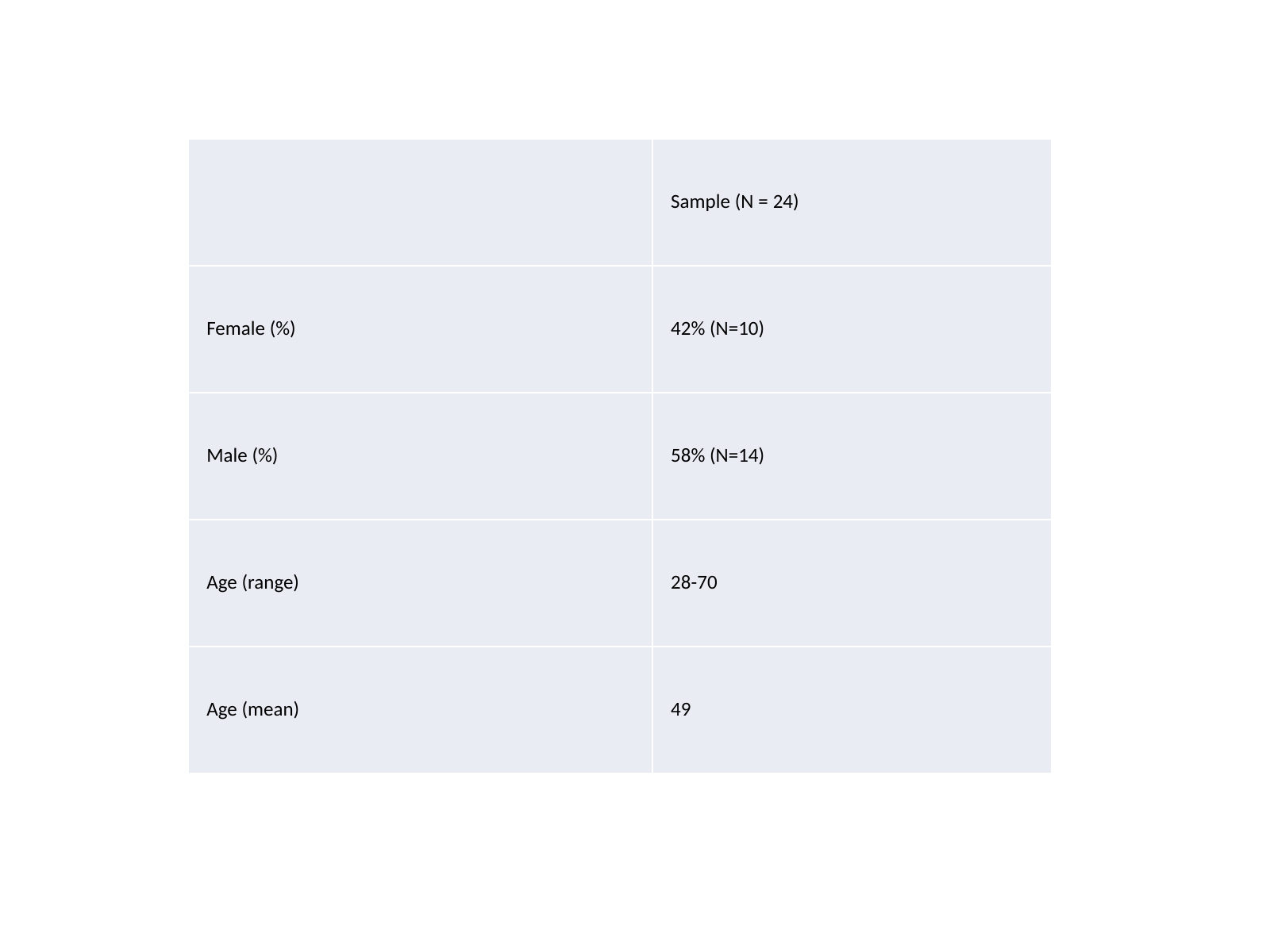

| | Sample (N = 24) |
| --- | --- |
| Female (%) | 42% (N=10) |
| Male (%) | 58% (N=14) |
| Age (range)​ | 28-70​ |
| Age (mean) | 49 |

## Slide 3
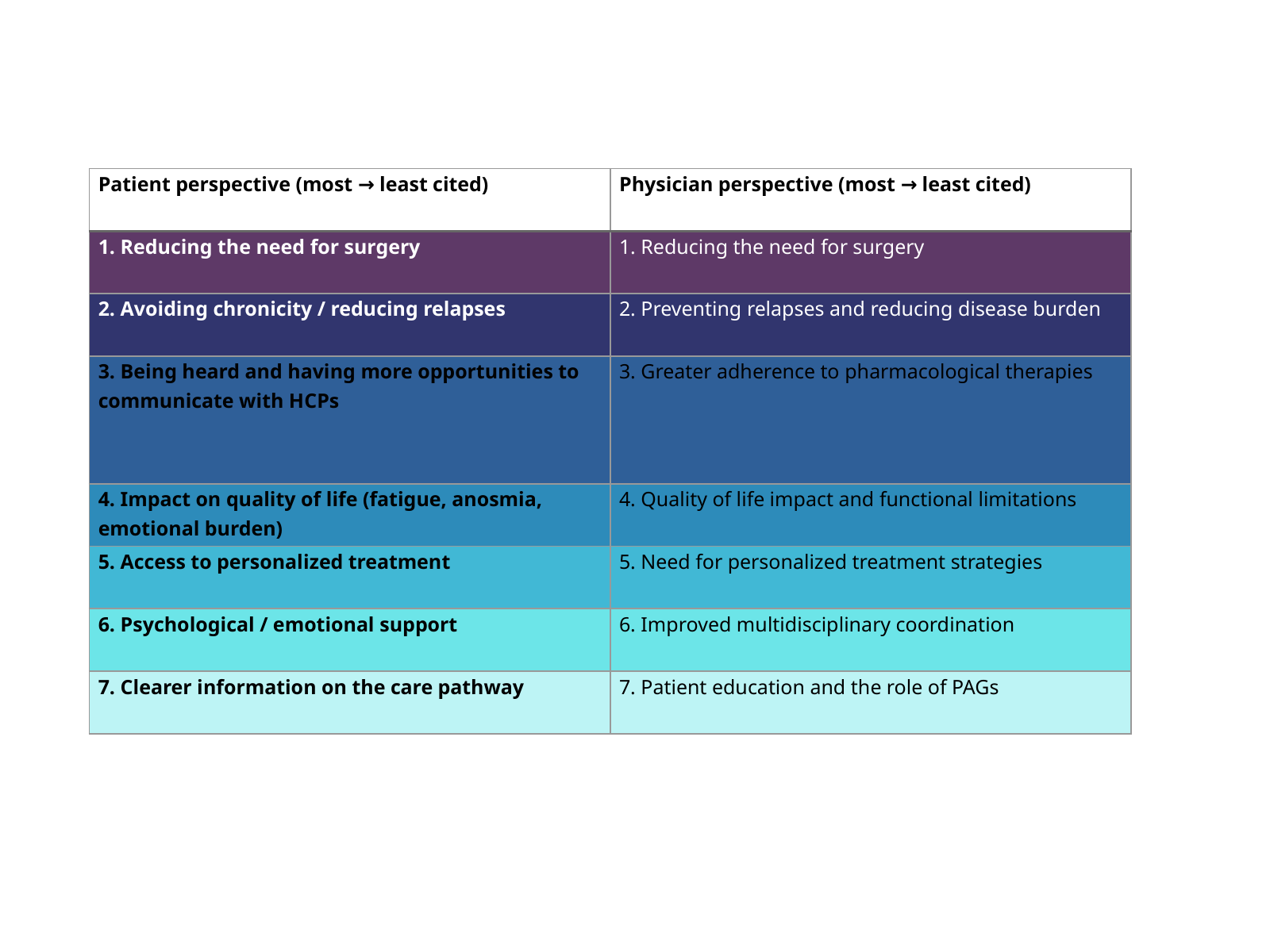

| Patient perspective (most → least cited) | Physician perspective (most → least cited) |
| --- | --- |
| 1. Reducing the need for surgery | 1. Reducing the need for surgery |
| 2. Avoiding chronicity / reducing relapses | 2. Preventing relapses and reducing disease burden |
| 3. Being heard and having more opportunities to communicate with HCPs | 3. Greater adherence to pharmacological therapies |
| 4. Impact on quality of life (fatigue, anosmia, emotional burden) | 4. Quality of life impact and functional limitations |
| 5. Access to personalized treatment | 5. Need for personalized treatment strategies |
| 6. Psychological / emotional support | 6. Improved multidisciplinary coordination |
| 7. Clearer information on the care pathway | 7. Patient education and the role of PAGs |

## Slide 4
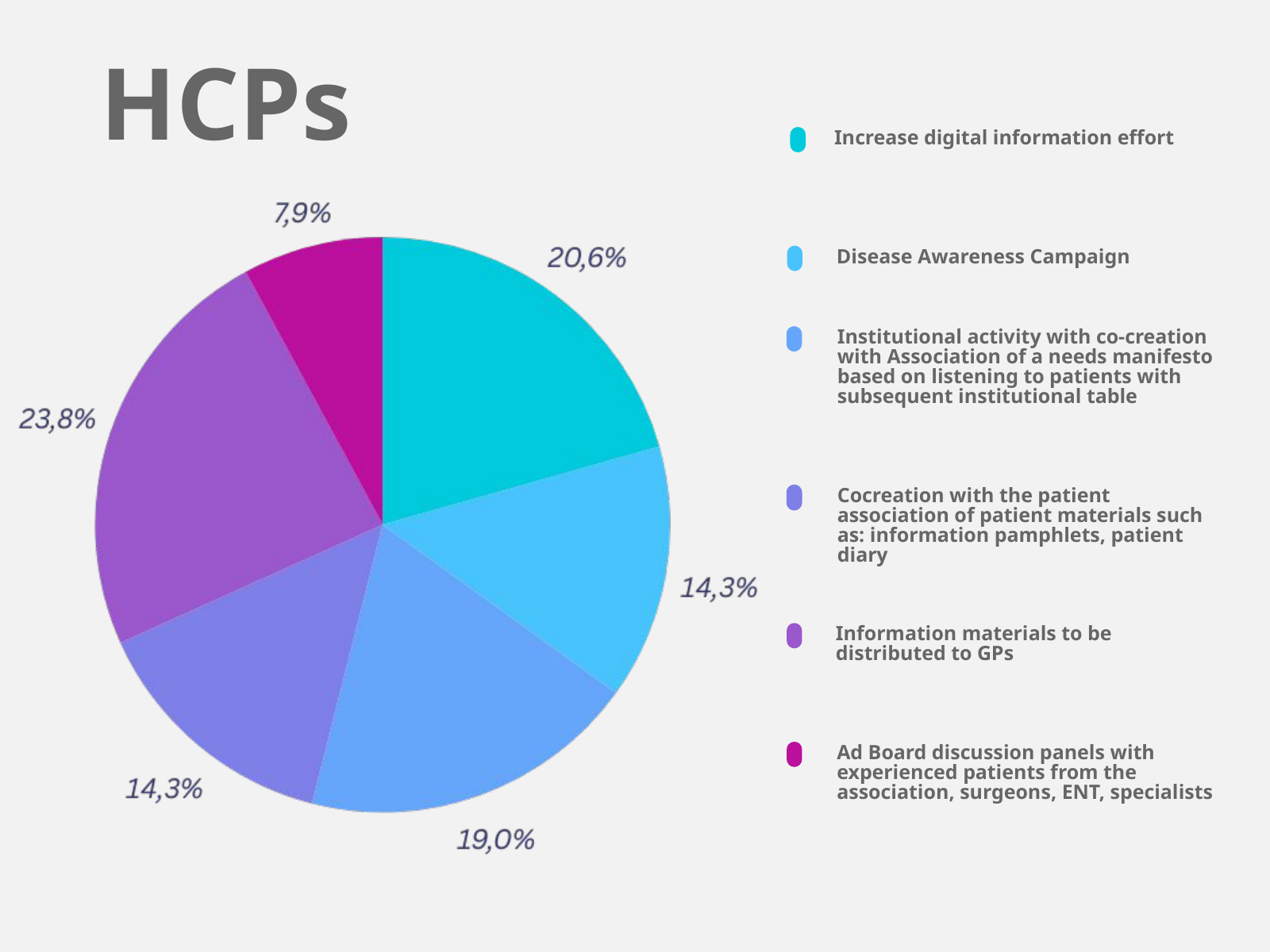

HCPs
Increase digital information effort
Disease Awareness Campaign
Institutional activity with co-creation with Association of a needs manifesto based on listening to patients with subsequent institutional table
Cocreation with the patient association of patient materials such as: information pamphlets, patient diary
Information materials to be distributed to GPs
Ad Board discussion panels with experienced patients from the association, surgeons, ENT, specialists

## Slide 5
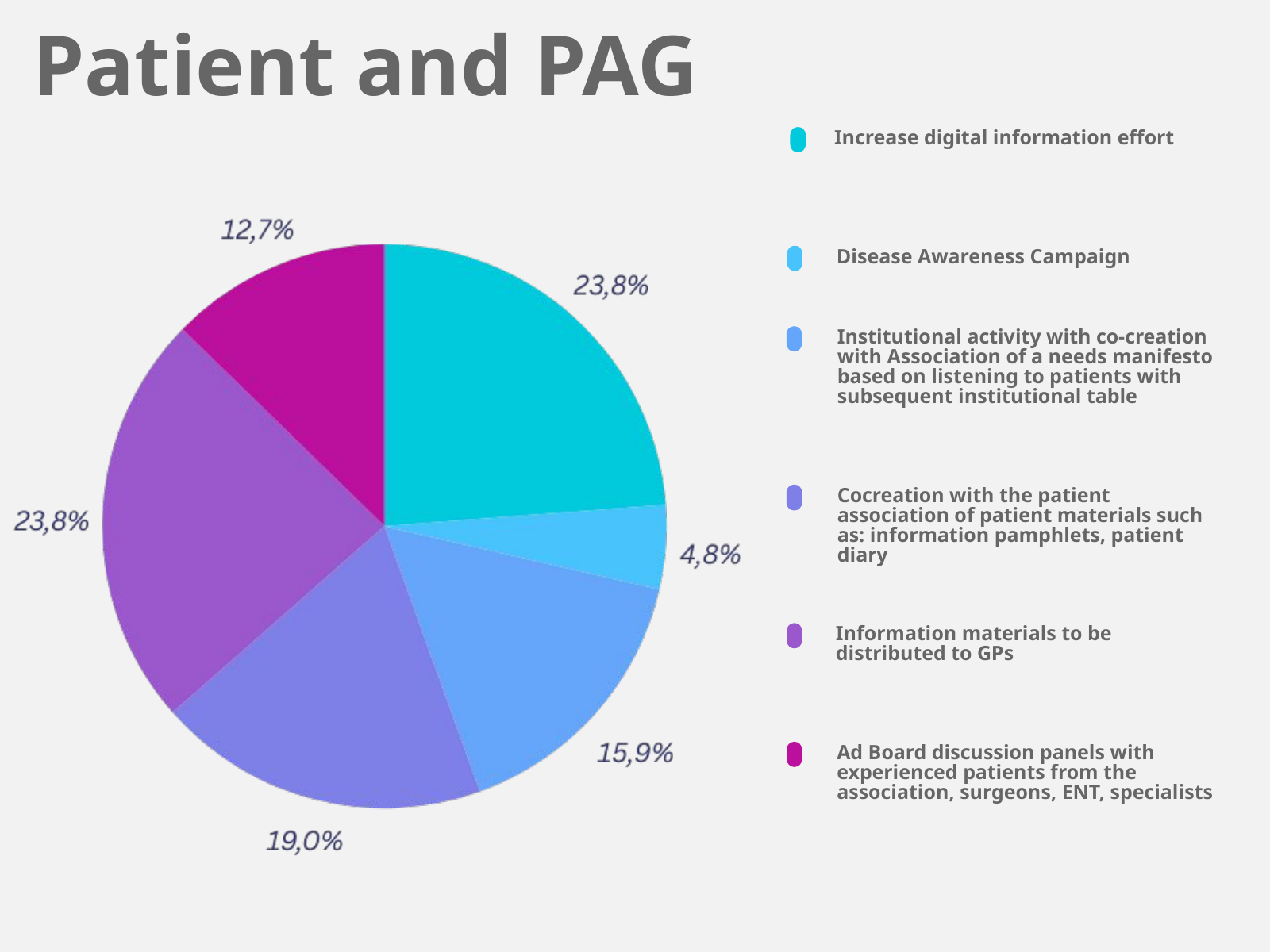

Patient and PAG
Increase digital information effort
Disease Awareness Campaign
Institutional activity with co-creation with Association of a needs manifesto based on listening to patients with subsequent institutional table
Cocreation with the patient association of patient materials such as: information pamphlets, patient diary
Information materials to be distributed to GPs
Ad Board discussion panels with experienced patients from the association, surgeons, ENT, specialists

## Slide 6
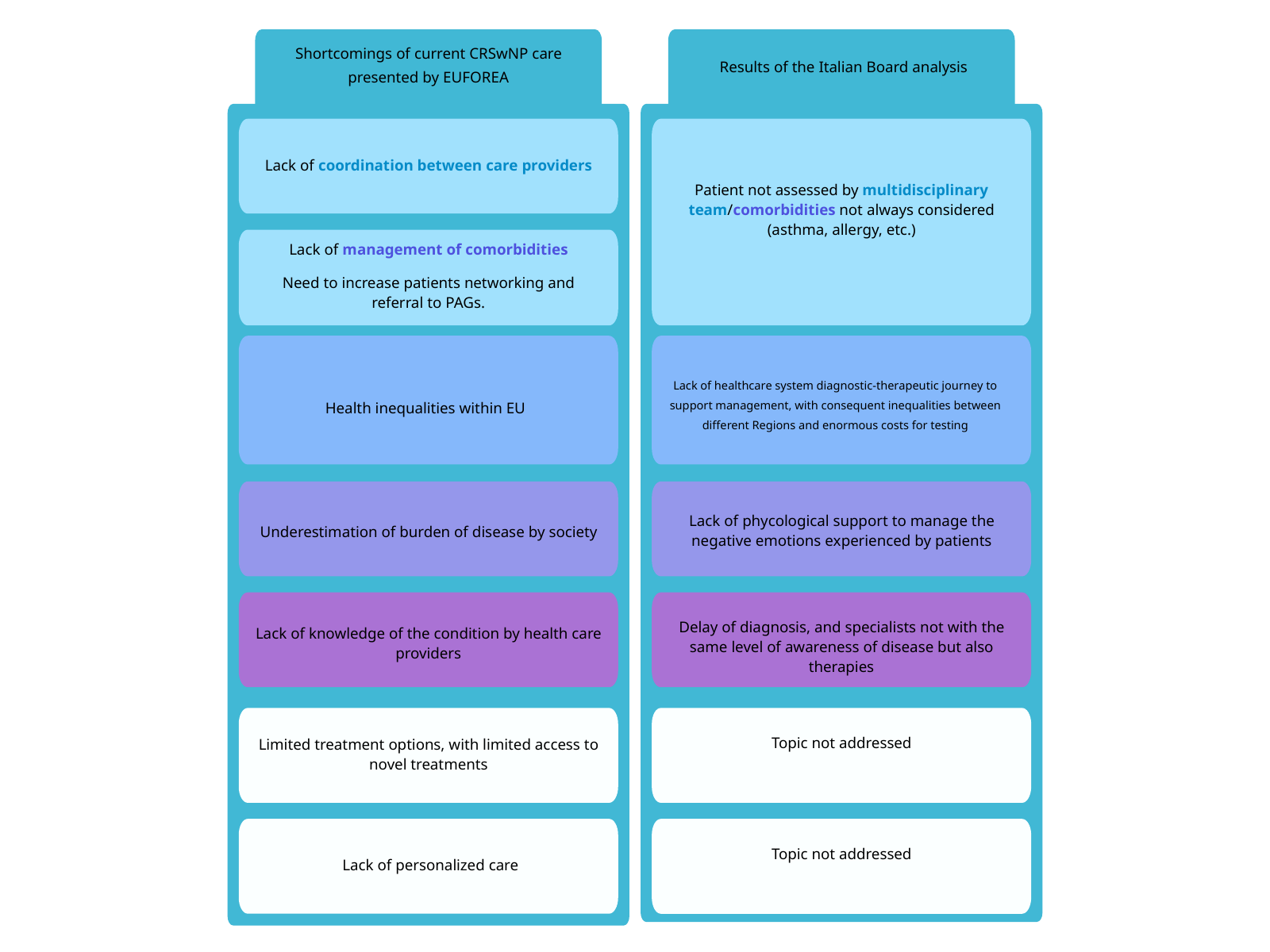

Shortcomings of current CRSwNP care presented by EUFOREA
 Results of the Italian Board analysis
Patient not assessed by multidisciplinary team/comorbidities not always considered (asthma, allergy, etc.)
Lack of coordination between care providers
Lack of management of comorbidities
Need to increase patients networking and referral to PAGs.
Lack of healthcare system diagnostic-therapeutic journey to support management, with consequent inequalities between different Regions and enormous costs for testing
 Health inequalities within EU
Lack of phycological support to manage the negative emotions experienced by patients
Underestimation of burden of disease by society
Lack of knowledge of the condition by health care providers
Delay of diagnosis, and specialists not with the same level of awareness of disease but also therapies
Topic not addressed
Limited treatment options, with limited access to novel treatments
Topic not addressed
 Lack of personalized care
